# Potential pitfalls when denoising resting state fMRI data using nuisance regression

**DOI:** 10.1016/j.neuroimage.2016.12.027

**Published:** 2017-07-01

**Authors:** Molly G. Bright, Christopher R. Tench, Kevin Murphy

**Affiliations:** a Sir Peter Mansfield Imaging Centre, School of Medicine, University of Nottingham, Nottingham, United Kingdom; b Division of Clinical Neurosciences, School of Medicine, University of Nottingham, Nottingham, United Kingdom; c Cardiff University Brain Research Imaging Centre (CUBRIC), School of Psychology, Cardiff University, Cardiff, United Kingdom; d CUBRIC, School of Physics and Astronomy, Cardiff University, Cardiff, United Kingdom

**Keywords:** Resting state, fMRI, Noise correction, Nuisance regression, Connectivity

## Abstract

In resting state fMRI, it is necessary to remove signal variance associated with noise sources, leaving cleaned fMRI time-series that more accurately reflect the underlying intrinsic brain fluctuations of interest. This is commonly achieved through nuisance regression, in which the fit is calculated of a noise model of head motion and physiological processes to the fMRI data in a General Linear Model, and the “cleaned” residuals of this fit are used in further analysis. We examine the statistical assumptions and requirements of the General Linear Model, and whether these are met during nuisance regression of resting state fMRI data. Using toy examples and real data we show how pre-whitening, temporal filtering and temporal shifting of regressors impact model fit. Based on our own observations, existing literature, and statistical theory, we make the following recommendations when employing nuisance regression: pre-whitening should be applied to achieve valid statistical inference of the noise model fit parameters; temporal filtering should be incorporated into the noise model to best account for changes in degrees of freedom; temporal shifting of regressors, although merited, should be achieved via optimisation and validation of a single temporal shift. We encourage all readers to make simple, practical changes to their fMRI denoising pipeline, and to regularly assess the appropriateness of the noise model used. By negotiating the potential pitfalls described in this paper, and by clearly reporting the details of nuisance regression in future manuscripts, we hope that the field will achieve more accurate and precise noise models for cleaning the resting state fMRI time-series.

## Highlights

• Pre-whitening should be used in nuisance regression of resting state fMRI data.
• Incorporating temporal filtering into GLM accounts for changed degrees of freedom.
• Temporally shifting of nuisance regressors is sometimes warranted and advised.
• Optimal temporal shifts may not be reliably estimated from resting state data.
• Established nuisance regressors should be routinely assessed in new datasets.

## 1 Introduction

When characterising or quantifying brain activity using fMRI data, it is essential that we differentiate the true signal of interest from other noise-related fluctuations. Methods for isolating activation in task-based fMRI, where an experimental stimulus can be modelled, are well-developed and validated. However, this differentiation is more challenging in resting state fMRI, where we have no model of the intrinsic brain activity of interest. Instead, in these experiments we approach analysis from the other direction: although we cannot model the activation, we can measure and model numerous noise sources. Any signal not accounted for by our noise model becomes the *de facto* representation of intrinsic brain activity. The method by which we define and remove noise fluctuations is therefore integral to our interpretation of resting state fMRI and functional connectivity. Confounding noise sources include scanner artefacts (e.g., drift), head motion with related spin history effects, and numerous physiological factors related to cardiac and respiratory processes (Murphy et al., 2013). Extensive research has focused on how to measure, model, and remove noise, as reflected by several of the other articles in this special issue. There is evidence that current denoising is insufficient, and there remains a bias in connectivity values due to noise confounds (Liu, 2016, Murphy et al., 2013); the temptation is then expansion of our noise model to address this systematic bias. However, as the noise model is expanded, we are more likely to encounter the pitfalls of using linear regression to accurately denoise data. For example, we have shown that nuisance regression results in incorrect classification of intrinsic signal fluctuations in multiple brain networks as “noise” (Bright and Murphy, 2015). This problem is compounded as the size of our noise model is increased, resulting in a real concern that our efforts to remove confounding noise fluctuations may also result in unintentional removal of our signal of interest. In this paper, we highlight some of the potential pitfalls encountered when applying and interpreting linear models in the context of resting state fMRI. Many researchers are likely already aware of the issues at hand, however it is not clear from the literature whether these problems are appropriately negotiated across the field. We explain the requirements and assumptions of the general linear model, and assess whether they are met during resting state denoising. We show how existing pre-whitening techniques can be applied to enable valid statistical inference of the model fit, using real resting state fMRI data to demonstrate the impact of pre-whitening on the variance removed by both real and simulated noise models. The temporal properties of individual nuisance regressors, both inherent to the noise source and the result of pre-processing steps (e.g., inherent spectral properties, temporal filtering, temporal shifting), can artificially inflate the amount of variance removed during regression; we characterise these potential confounds and discuss ways in which they can be taken into account. Finally, we present our recommendations and highlight areas of future research that we hope will improve how we, as a field, approach the cleaning and interpretation of resting state fMRI data.

## 2 Theory

### 2.1 The general linear model

The basic form of the general linear model (GLM) is

Y=Xβ+e

where the statistical assumptions and requirements are as follows:

1. The system must be linear
2. ***X*** is a design matrix containing linearly independent explanatory variables
3. ***Y*** is (linearly) dependent on the explanatory variables contained in ***X*** through the weights ***β***; these weights are the model parameters.
4. The model is complete, such that the explanatory variables explain the deterministic variance in ***Y*** leaving only residual errors. These errors should ideally be estimates of ***e***.
5. The true errors, ***e***, are independent and identically distributed (i.i.d.) and have constant variance (heteroscedasticity). The estimates of ***e*** have similar requirements, although they are not strictly independent due to the parameters ***β***.
  If inference on the model parameters is desired, there is an additional requirement:
6. In addition to being i.i.d., the errors must be normally distributed: e~N(0,Σ).

It is typically expected that the error, $e\sim N\left(0,\sigma^{2}I\right)$, where ***I*** is the identity matrix. In this case, parameter estimation and inference is by *t*-tests, and a test of the overall model fit is analytic. However, if the errors violate the assumptions, statistical inference using the GLM may not be valid; in the absence of a-priori knowledge of the distribution of the errors, an alternative non-parametric method may be used at the cost of some statistical power (Nichols and Holmes, 2002).

### 2.2 GLM requirements for nuisance regression in resting state fMRI

We have discussed how such statistical inference is necessary to the systematic assessment and refinement of our noise model in resting state fMRI, and that it is critical we determine whether the statistical requirements listed above are met in the context of nuisance regression.

Typically, the design matrix ***X*** is formed from nuisance regressors reflecting head motion and physiologic noise sources, while the observations ***Y*** are the resting Blood Oxygenation Level Dependent (BOLD) fMRI time-series data, often with basic pre-processing applied (e.g., motion correction). The denoised resting state time-series is defined as the residual of the model fit. In the field of functional brain connectivity we hypothesize that these time-series contain coherent signal fluctuations, reflecting coupled neural activity across different brain regions.

In this context, we encounter several issues affecting the GLM:

• The explanatory variables, which are the nuisance regressors, are not typically linearly independent. Motion of the head during scanning may impact all translation and rotation parameters in a correlated way, and changes in heart rate and arterial blood gases may also be coupled due to shared physiologic mechanisms.
• The model is not complete: the underlying intrinsic brain fluctuations that are ultimately of interest are not modelled.
• Because the residual errors are the *de facto* BOLD signal of interest, we have the axiomatic problem that these “errors” are non-white.

The use of the GLM for nuisance regression in resting state fMRI is clearly in conflict with the statistical assumptions listed above.

The first concern is that the nuisance regressors in the noise model are not completely linearly independent and may exhibit shared variance. However, providing that the nuisance regressors are not linear combinations of each other, a solution to the GLM can be obtained. The difficulty will arise later, when signal variance may be arbitrarily attributed to temporally similar nuisance regressors. Thus, while the covariance inherent in the explanatory variables does not preclude the use of the GLM, it makes the relative contribution of specific nuisance regressors more difficult to interpret (e.g., during model selection).

However, the incompleteness of our model (and, as a direct consequence, the non-white properties of the residual errors) directly calls into question the validity of all inference in the GLM.

### 2.3 Achieving valid statistical inference via pre-whitening

There exist numerous techniques for addressing the problem of non-white residuals in the GLM. Because the true autocorrelations of the residuals are not known, filtering may be employed to shape (“pre-colour”) the residuals into something that is known (Smith et al., 2004). Alternatively, pre-whitening estimates the autocorrelation in the residuals and removes it (Bullmore et al., 1996, Woolrich et al., 2001). Numerous pre-whitening tools are readily available in the major fMRI analysis packages, and are typically recommended when using a GLM to model task-activation fMRI data (Smith et al., 2004).

For example, if assuming the residuals can be characterized by an auto-regressive ***AR(p)*** model, then we must solve the equation

$$Y_{t}=X_{t}\beta +e_{t}$$

where the residuals ***e***_***t***_ are described as an AR(p) process

$$e_{t}=\Sigma_{i=1}^{p}\gamma_{i}e_{t-i}+\varepsilon_{t}$$

and ***ε*** represents i.i.d. and normally distributed errors.

An algorithmic approach for estimating the unknown model fit parameters ***β*** and the unknown ***AR(p)*** time-series parameters ***γ*** is as follows (Bullmore et al., 1996; Cochrane and Orcutt, 1949):

1. Estimate ***β*** using ordinary least squares and extract the residuals ***e***.
2. Fit the residuals with an ***AR(p)*** model (estimate the ***γ***_***i***_ parameters)
3. Redo the ordinary least squares fitting on a modified model.

The modified model is defined as

Y′=X′β+e′,

where

$${Y\prime }_{t}=Y_{t}-\Sigma_{i=1}^{p}\gamma_{i}Y_{t-i}$$

and

$${X\prime }_{t}=X_{t}-\Sigma_{i=1}^{p}\gamma_{i}X_{t-i}$$

This procedure can be iterated if needed, or adjusted to incorporate more complex models such as Auto-Regressive Moving Average (ARMA) or Auto-Regressive Integrated Moving Average (ARIMA) models.

In denoising resting state fMRI data, our residuals are our signal of interest; thus in pre-whitening we are effectively modelling the underlying intrinsic brain fluctuations as an autocorrelative process. Exactly how to model these fluctuations is non-trivial. In task-activation fMRI analysis, the residuals consist of unmodeled physical or physiological noise sources and are generally considered to be well modelled by an ***AR*****(*****p*****)** process. It is not clear whether this model would sufficiently characterise resting state fluctuations, or whether ARMA, ARIMA, or other models would be required. The order of the autoregressive model ***p*** (the maximum number of lags to consider) may also depend on scanning parameters. Much of the intrinsic autocorrelation in fMRI time-series comes from the sluggish haemodynamic response that produces the BOLD signal following an underlying neuronal event. If the fMRI sampling frequency is increased (TR is reduced), a greater number of lags may need to be included in the model (Arbabshirani et al., 2014).

Thus it is not the aim of this paper to prescribe specific pre-whitening methods, but rather to demonstrate that some form of pre-whitening, confirmed to be appropriate for a given study, should be employed during nuisance regression to enable interpretation and assessment of the noise model.

### 2.4 Assessing individual nuisance regressors

After ensuring that the fit statistics estimated in the GLM fit are valid via pre-whitening, it is important to also consider whether any other factors, either inherent to the data or created by pre-processing steps, may create bias in these statistics.

#### 2.4.1 Temporal filtering

A motivating factor for temporal filtering is that it hypothetically differentiates signal and noise frequency bands. Given that we are directly modelling multiple noise sources, this is greatly redundant. There is also evidence that the intrinsic brain fluctuations may be broadband in nature, extending up to 0.8 Hz (Chen and Glover, 2015; Lee et al., 2013; Niazy et al., 2011).

If filtering is performed, it must be either

a) applied following GLM fitting
b) applied prior to GLM fitting, and applied identically to both the noise model and the fMRI data to avoid the re-introduction of filtered frequencies (Hallquist et al., 2013)
c) applied during GLM fitting, by including additional regressors into the noise model (e.g., polynomials, sines and cosines, etc) (Hallquist et al., 2013, Jo et al., 2013).

**Fig. 1:**
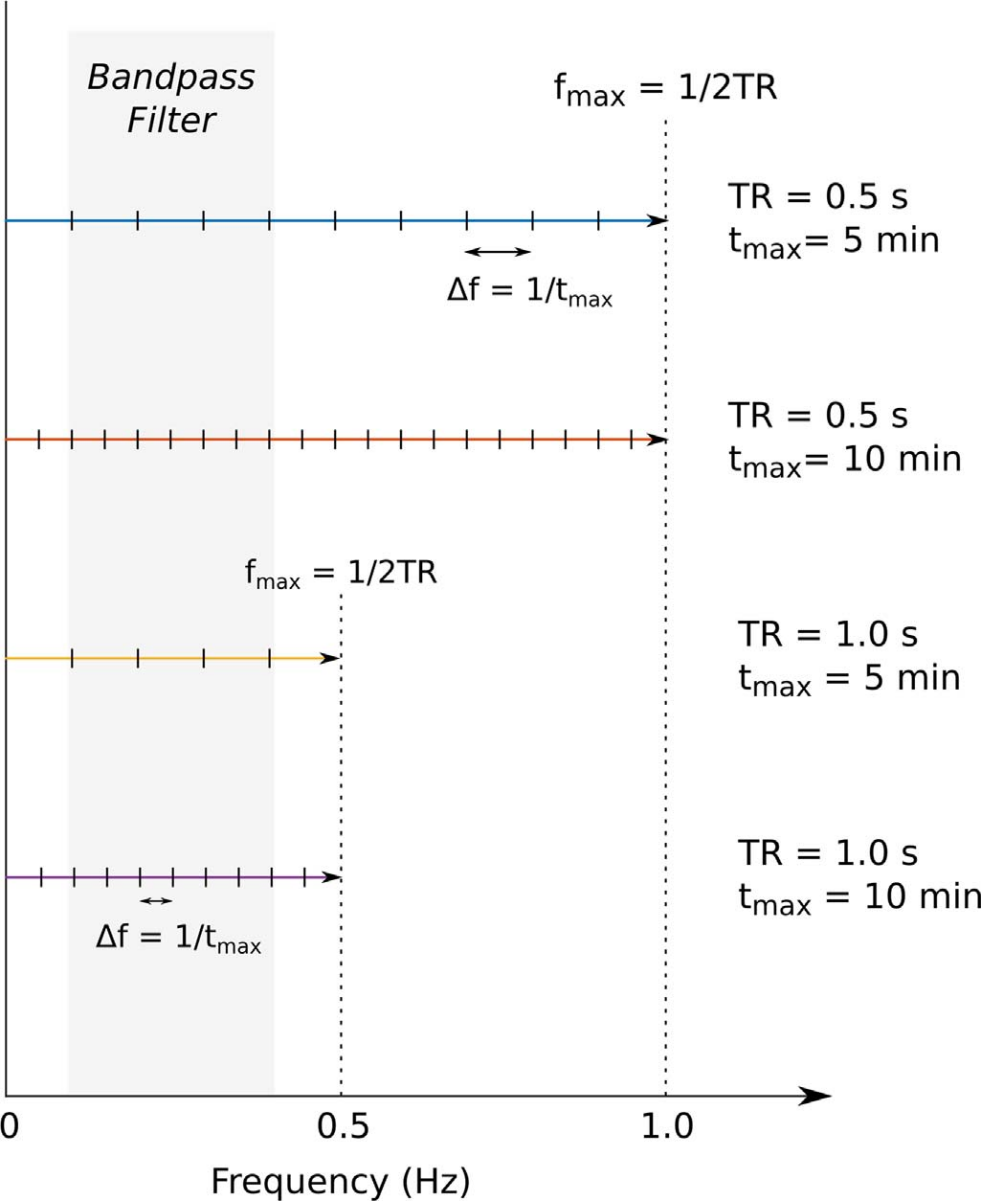
Schematic demonstrating how bandpass filtering influences the degrees of freedom in data of different durations and TRs. The Fourier transform of an fMRI time-series produces a frequency spectrum in which the maximum frequency is defined by the Nyquist frequency 1/2TR and the frequency spacing is determined by the total duration of the scan. The degrees of freedom remaining after applying a bandpass filter is dependent on the duration of the scan, whereas the proportion of degrees of freedom remaining in the filtered data relative to the unfiltered data depends on TR.

In addition to the method of applying filtering, it is also critical to consider how temporal filtering affects the degrees of freedom available in the data, and how this impacts the statistical tests on GLM fitting parameters. It is perhaps easiest to consider temporal filtering in frequency space (the Fourier transform of the time-series). Fig. 1 is a schematic showing the degrees of freedom available before and after applying a bandpass filter. In frequency space, the maximum frequency sampled is the Nyquist frequency, $f_{\max}=1/2TR$, and the frequency spacing is the inverse of the total scan duration, $\Delta f=1/t_{\max}$. A bandpass filter of 0.01–0.2 Hz applied to 5 minutes of data acquired at a TR of 1 s will reduce the degrees of freedom from 150 to ∼57, whereas a bandpass filter of 0.01–0.1 Hz reduces this even further to 27.

**Fig. 2:**
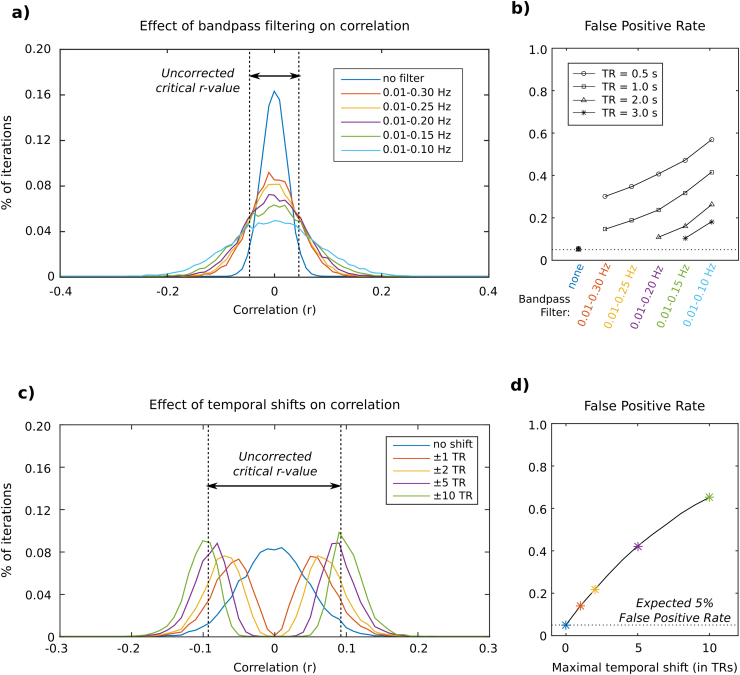
Simulated toy examples demonstrating the effect of temporal filtering and temporal shifts on the correlation between randomly generated time-series. a) Ten thousand pairs of random, normally distributed time-series (15 minutes of data at TR=1 s) were generated and bandpass filtered; the correlation between each time-series pair was calculated, and the distribution of the measured Pearson correlation coefficient *r* is plotted. The critical r-value associated with a threshold of p=0.05 is indicated (dashed lines). When the data are filtered, the normal distribution of r widens, causing a greater number of “false positive” significant correlations greater than this threshold value. b) The percentage of correlation values (out of 10,000) with an absolute value greater than the critical r-value is plotted for each bandpass filter, showing how filtering increases this “False Positive Rate.” Note that while increasing TR reduces the impact of bandpass filtering on the False Positive Rate, it also reduces the dataset's degrees of freedom for a given length of scan, which is not represented here. c) Using the unfiltered simulated data, one time-series of each pair was allowed to shift forwards and backwards in time (using the *xcorr* function in Matlab) and the (absolute) maximum correlation across the shifts was recorded. Histograms of the resulting maximum correlation values are plotted for different ranges of temporal shift considered (no shift, ±1TR, ±2TR, ±5TR, ±10TR). As a greater number of temporal shifts is considered, the distribution of the maximum r-value changes from a normal distribution to a bimodal distribution. d) The number of r-values above the critical r-value was counted and is plotted as a function of maximum temporal shift, showing over a ten-fold increase in the False Positive Rate when 20 shifted variants of one time-series are considered.

If filtering is applied *during* the GLM, each new term added to the noise model removes one degree of freedom, and the statistical tests would reflect this change in the available data. Concern would only arise if extensive amounts of additional filtering terms were added to the model such that any linear combination of them were collinear with any linear combination of noise regressors, causing the matrix to become singular. However, if filtering is applied to the data and noise model *before* GLM fitting, the correlation statistics will be artificially inflated (Fig. 2a, b). We refer you to the literature for a thorough description of how to correct t-statistics and p-values in this scenario (Davey et al., 2013); note, however, that this type of correction is only useful in correctly testing the significance of a given nuisance regressor in the noise model; it does not correct the fMRI variance removed by that regressor in the GLM.

#### 2.4.2 Temporal shifting

There are numerous instances where we may expect some temporal lag between a nuisance regressor and the corresponding fluctuations in fMRI signal. This is particularly true in our modelling of physiological noise, which may be lagged due to delays in the measurement (e.g., end-tidal gas measurements are delayed by slow breathing and potentially long sample lines) as well as delays inherent to the physiologic process (e.g., different vascular pathways and properties may cause brain regions to respond to changes in arterial gases at different times) (Bright et al., 2009).

It is therefore desirable to optimise any temporal offset between our nuisance regressors and the fMRI data to achieve the most “accurate” noise model by allowing the regressor to be shifted forwards and backwards in time. One option is to include many of these shifted variants of the original nuisance regressor in the noise model, removing any fMRI fluctuations that correspond to any of these temporal lags (e.g., “multi-lagged” approach presented in (Bianciardi et al., 2009) uses 8 shifted variants of the original respiratory noise regressor); however, this potentially reduces the degrees of freedom available in the data unnecessarily. An alternative two-step option is to identify the optimal shift in the regressor that results in maximal correlation with the fMRI data, and then use only that regressor in the model. For example, using the RIPTiDe technique, 61 temporally shifted variants of a physiological regressor were tested separately, and the variant with maximum correlation was identified for every voxel for use in further analysis (Tong et al., 2011).

The crucial point in both scenarios is that considering shifted variants is practically guaranteed to increase the variance explained by the nuisance regressor, even when it reflects a spurious relationship. In Fig. 2c, d we demonstrate this using randomly generated time-series: when the time-series are allowed to shift forwards and backwards in time, the maximal correlation at an “optimal” shift follows a bimodal rather than normal distribution. The new distribution is clearly biased towards stronger correlation values. In nuisance regression, this will equate to artificially “significant” relationships observed between the regressor and the data when none may exist, and increases in variance removed from the fMRI data at random.

This issue can be viewed as a multiple comparisons problem: each variant of the regressor, shifted forwards or backwards in time, results in another correlation test. The Šidák correction adjusts p-values for multiple independent tests (Šidák, 1967). Assuming a significance threshold for correlation, α, the Šidák corrected threshold is

$$\alpha_{sidak}=1-{\left(1-\alpha \right)}^{\frac{1}{m}}$$

where *m* is the number of tests (or number of regressor variants) considered. For example, in the aforementioned case where 61 variants of the regressor were considered, the maximal correlation should have a p-value less than 8.4×10^−4^ (Z>3.1) to be deemed statistically significant at α=0.05, and a p-value less than 1.6×10^−4^ (Z>3.6) to be deemed statistically significant at α=0.01. Note that this correction is sufficient for normally distributed random time-series, but further corrections may be needed if testing time-series with autocorrelative properties (Arbabshirani et al., 2014).

In the case of shifted nuisance regressors, the time-series are not independent. Thus, the Šidák correction is a conservative approach for accounting for temporal shifting in a noise model. Alternative methods for correcting correlation statistics for multiple temporal shifts may be found in the literature (Shmueli et al., 2007).

Ultimately, temporal shifts are often appropriate, however the correlation identified at an optimal temporal shift of the nuisance regressor should exceed a significance threshold that has been properly corrected for multiple tests. This will be demonstrated using real data examples in the next sections.

## 3 Methods

Although the statistical theory described above and the toy examples in Fig. 2 aptly demonstrate the fundamental statistical concepts involved in nuisance regression, it is important to assess how these concepts manifest in real fMRI data. We examine the model fit parameters of nuisance regression in resting state fMRI data acquired in a small cohort with fairly typical acquisition parameters. Based on our observations, we will make recommendations for how the denoising of similar datasets may be best approached.

### 3.1 Data acquisition

Resting state fMRI data were acquired as part of a prior study (Bright and Murphy, 2013a). Twelve healthy subjects (aged 32±6 years, 5 female) were scanned using a 3 T GE HDx scanner (Milwaukee, WI, USA) equipped with an 8-channel receive head coil. An eyes-open resting state scan lasting 5.5 min was acquired using a T_2_^*^-weighted gradient-echo echo-planar imaging sequence (TR/TE=2000/35 ms; FOV=22.4 cm; 35 slices, slice thickness=4 mm; resolution=3.5×3.5×4.0 mm^3^, 165 volume acquisitions). The data were motion corrected, corrected for slice timing differences, and brain extracted (AFNI, http://afni.nimh.nih.gov/afni (Cox, 1996)). The first 5 volumes, during which steady-state magnetisation was not yet achieved, were removed.

### 3.2 True nuisance regressors

Cardiac pulsations were monitored using the scanner finger plethysmograph; the timing of each pulse was recorded and beat-to-beat heart rate was calculated. Expired CO_2_ content was monitored during scanning via a nasal cannula (AEI Technologies, PA, USA) and end-tidal CO_2_ (P_ET_CO_2_) values were extracted using bespoke software (MathWorks, Natick, MA, USA). The heart rate and P_ET_CO_2_ data were smoothed using a CRF and HRF function, respectively (Chang et al., 2009) before inclusion in our noise model.

The six head motion regressors derived during motion correction (x-, y-, z-translations and pitch, roll, yaw rotations) were also included in our noise model. Combined with the above heart rate and P_ET_CO_2_ regressors, these are referred to as the “true” nuisance regressors.

### 3.3 Simulated noise models

To form our null hypothesis, we also analysed two additional noise models consisting of nuisance regressors that are unrelated to the fMRI data. First, we considered the nuisance regressors from a different subject, which may have similar time-series properties but are theoretically independent of the fMRI data from a different scan. For simplicity, in the noise model for subject *N*, we used the regressors of subject *N*+*1*. Second, we considered phase-randomised versions of the true nuisance regressors. This procedure was used previously (Bright and Murphy, 2015) to simulate new regressors with the same frequency content as the original ones. Note that in both scenarios we do not enforce orthogonality with the true regressors, and by chance there may be some similarity between the regressors across subjects or after phase randomisation.

### 3.4 Model fitting

The variance associated with the 8-regressor noise model (either from true regressors, regressors from another subject, or simulated regressors using phase-randomisation) was removed from the functional fMRI data of each subject using the 3dDeconvolve and 3dREMLfit programmes in AFNI. 3dDeconvolve is a standard GLM programme, whereas 3dREMLfit uses pre-whitening to account for serial autocorrelation in the GLM residuals, modelling them as an ARMA(1,1) process using Restricted Maximum Likelihood (REML). In all cases, additional parameters were included to detrend the data, removing baseline values and linear/quadratic trends during the fitting rather than applying temporal filters prior to fitting. As described above, this approach was chosen to remove scanner drift without making assumptions about higher frequency signal contributions, and it was incorporated into the model to account for the impact on degrees of freedom.

In the 3dREMLfit results, the residuals were assessed for any remaining autocorrelation, and the cleaned fMRI time-series were calculated by subtracting the noise model fit from the original data. In all results, the R^2^ of the noise model (not including detrending terms) was extracted for each voxel, and an uncorrected threshold of p<0.05 was used to identify voxels where the fit was significant.

### 3.5 Temporal shifting

To ascertain the benefits and challenges of temporally shifting a given nuisance regressor, we examined the correlation between the mean %BOLD grey matter time-series from each dataset (calculated in (Bright and Murphy, 2013a)) and the associated P_ET_CO_2_ regressor across a range of temporal shifts. The P_ET_CO_2_ regressor was linearly interpolated to achieve an apparent temporal resolution of 0.2 s and demeaned; 81 shifted variants of the regressor were extracted, ranging from −4 s to +12 s in steps of 0.2 s, and the empty time-points were zero-filled. The correlation value between the fMRI time-series and the P_ET_CO_2_ regressor was calculated for all shifted variants.

The same analysis was performed using 10 simulated regressors (phase-randomised variants of the true P_ET_CO_2_ regressor).

Lastly, the relationship between fMRI data and the P_ET_CO_2_ regressor was determined, for the same range of temporal shifts, in a second dataset that contained 6 consecutive 20-second breath-holds (acquired during the same study as the resting state data (Bright and Murphy, 2013a)). By instructing participants to hold their breath, large increases in P_ET_CO_2_ levels (i.e., hypercapnia) were induced, and a large BOLD signal response was evoked.

The optimal temporal shift was identified in all cases. The optimal shifts were also calculated using the first and second halves of the data independently, and compared using Pearson's correlation coefficient for validation.

## 4 Results

### 4.1 Impact of pre-whitening on noise model fit

**Fig. 3:**
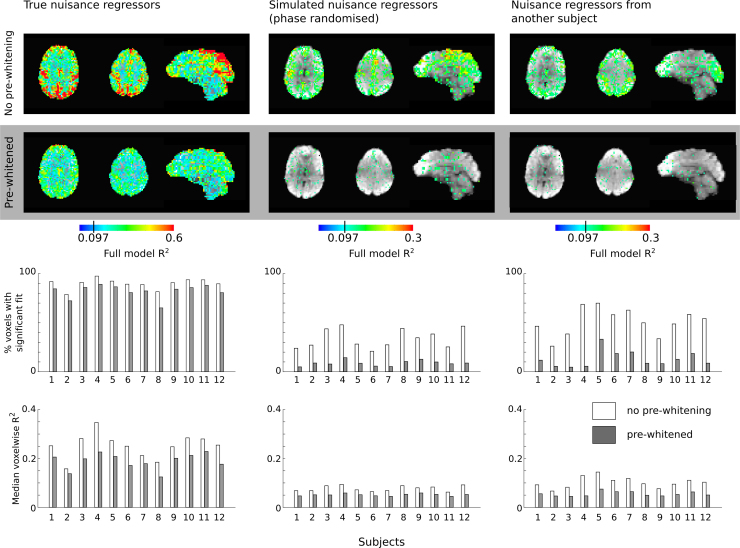
R^2^ maps estimating the percentage of variance removed by the total noise model (true, simulated, and from another subject) for an example subject, thresholded at R^2^>0.097 (p<0.05, uncorrected for multiple comparisons). Maps were generated using a General Linear Model without pre-whitening (AFNI 3dDeconvolve) or with pre-whitening steps (AFNI 3dREMLfit). The percentage of voxels in the brain where R^2^ exceeded the significance threshold, and the median R^2^ across all voxels, are plotted for each of the 12 subjects, with and without pre-whitening.

The results of the fitting procedure without (3dDeconvolve) and with (3dREMLfit) pre-whitening are summarised in Fig. 3. We observed that the incorporation of pre-whitening reduced the number of voxels where the model fit was significant, as well as the median voxelwise R^2^ for the noise model, for all noise models examined (paired t-tests, p<0.05, corrected for multiple comparisons). However, the size of this effect was dependent on whether the noise model consisted of true or unrelated nuisance regressors.

When the true noise model was assessed, the percentage of brain voxels where the model fit was significant was reduced by 9% due to pre-whitening, whereas it was reduced by 76% and 74% for the simulated and incorrect subject noise models, respectively (mean across subjects). The median voxelwise R^2^, approximately representing the amount of variance removed by the noise model, was also affected differently by pre-whitening across the three types of noise models, as shown in Fig. 3. R^2^ was reduced by 24%, 45%, and 33% for the true noise model, simulated noise model, and incorrect subject noise model, respectively. This observation is consistent with the hypothesis that pre-whitening will impact true relationships less than it will spurious relationships between the data and the noise model. However, the effects we observe cannot be easily interpreted without “ground-truth” knowledge of the true signal contributions of different noise sources to voxelwise data.

### 4.2 Temporal shifting of regressors

**Fig. 4:**
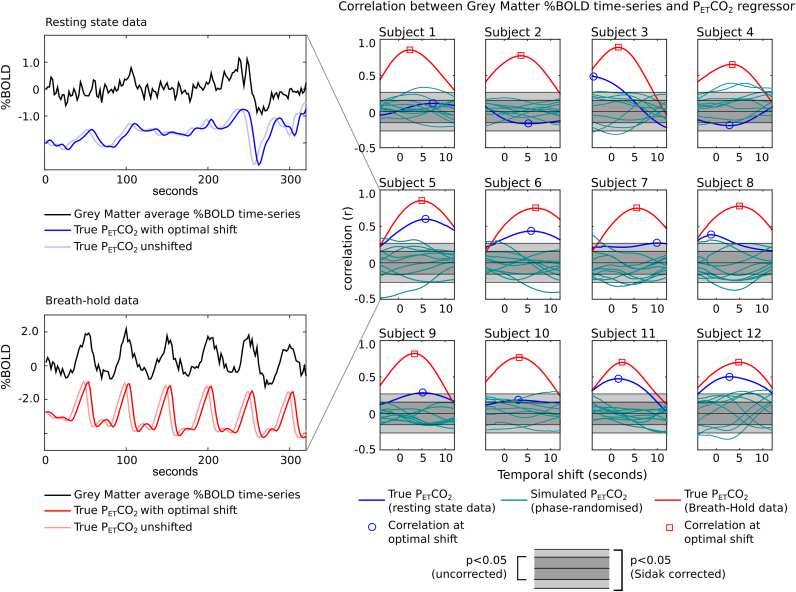
Optimisation of the temporal shift applied to the P_ET_CO_2_ regressor. For each subject, the correlation between P_ET_CO_2_ and the %BOLD time-series averaged across grey matter voxels is plotted as a function of the temporal shift, ranging from −4 to +12 seconds in steps of 0.2 s (81 shifts). The correlation values obtained in the resting state data for the true regressor (blue) and ten simulated phase-randomised regressors (cyan) are presented. The correlation values obtained in breath-hold data from the same subjects are also shown (red). The optimal shifts identified for the true regressors are indicated; the grey shaded regions represent the significance threshold of p<0.05 (r=0.16), and the same threshold after Šidák correction for 81 tests (p<6×10^−4^, r=0.27). The time-series and P_ET_CO_2_ regressors for the resting state data and breath-hold data of Subject 5 are provided as a reference.

The correlation between resting state data and P_ET_CO_2_ is plotted as a function of the temporal shift applied to the P_ET_CO_2_ regressor for each subject in Fig. 4. The equivalent results for simulated regressors, which have identical frequency content to the true regressor but which should be “unrelated” to the data, are also plotted.

**Fig. 5:**
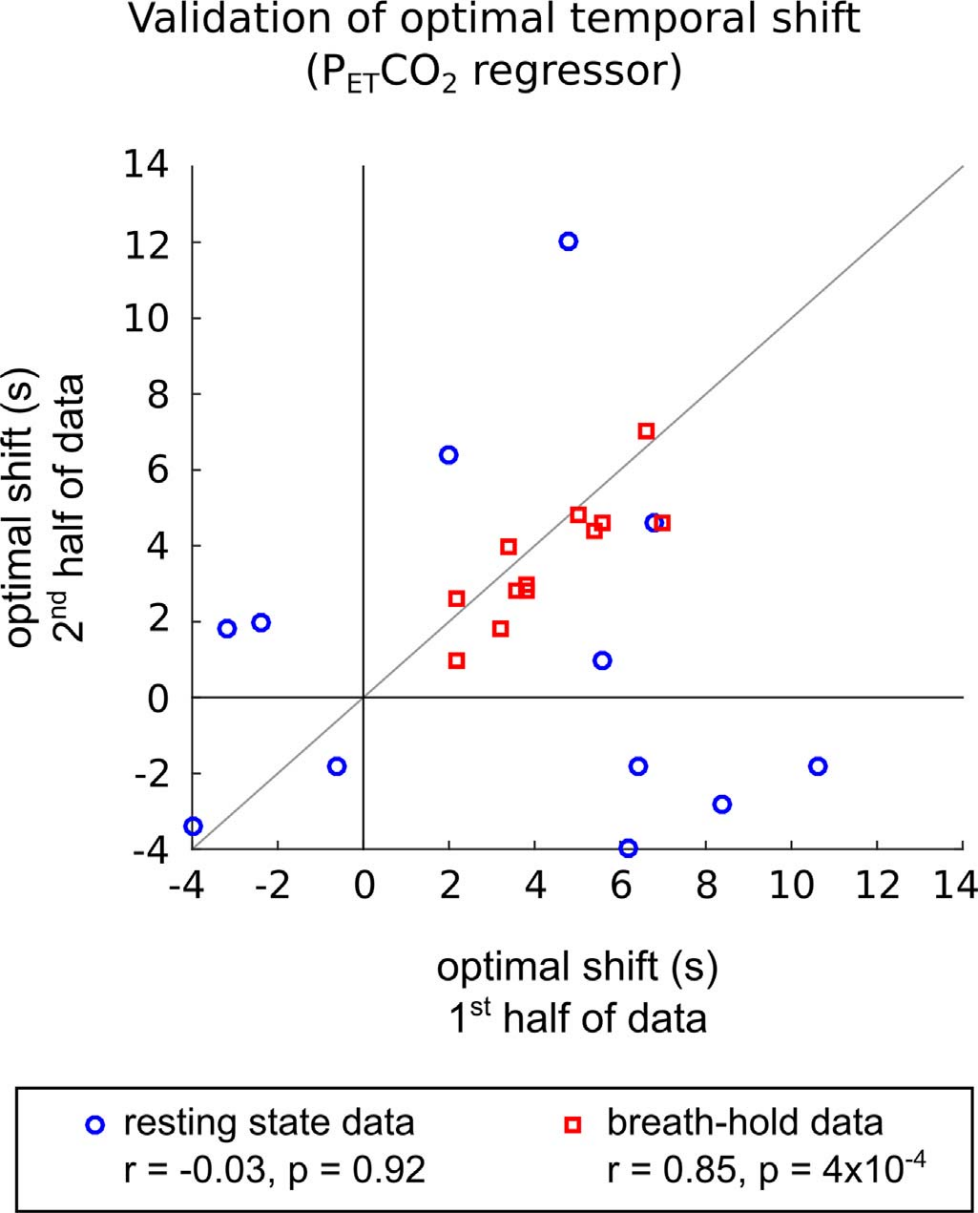
Validation of the optimal temporal shift of the P_ET_CO_2_ regressor. The optimal shift was defined as that which resulted in maximal (absolute) correlation between the regressor and grey matter average %BOLD time-series. The optimal shift was identified in the first and second halves of the data, for both the resting state (blue) and breath-hold (red) datasets, and then compared. There was no relationship between the shifts identified in the two halves of the resting state data (r=−0.03, p=0.92), suggesting that temporal shifting of the P_ET_CO_2_ regressor can not be accurately optimised in these data. By contrast, the breath-hold data revealed significantly correlated optimal shifts (r=0.86, p=4×10^−4^), demonstrating that the P_ET_CO_2_ regressor can (and should be) temporally shifted to best model the associated signal variance. Validation analysis, such as the correlation results presented here, should be used to confirm the robustness of any temporal shifts applied to nuisance regressors.

The strength of the measured correlation varies substantially across subjects, and there are several datasets in which the correlation is not significant for any temporal shift (significance threshold indicated with shaded grey region). There are also datasets (e.g., Subject 1) in which the fMRI time-series is more correlated with the simulated regressors than the true regressor at a given lag. The maximum absolute correlation is sometimes observed to be *negative* correlation (e.g., Subjects 2 and 4), which is not physiologically expected for the grey matter average time-series (Bright et al., 2013, Wise et al., 2004). Finally, the optimal temporal shift for the true P_ET_CO_2_ regressor varies greatly across subjects, sometimes not demonstrating a robust maximum within the temporal bounds considered (e.g., Subjects 3, 7 and 10). The results of the validation testing are presented in Fig. 5; there is no significant relationship observed between the optimal temporal shifts identified in the two halves of the fMRI dataset (r=−0.03, p=0.92). Combined, these observations indicate that the P_ET_CO_2_ regressor may not be robustly related to the resting state fMRI data, and in this circumstance it may not be appropriate to select an “optimal shift.”

By contrast, the breath-hold data show a significant positive correlation between the grey matter time-series and P_ET_CO_2_ regressor (Fig. 4, red lines), which reaches a clear local maximum at a positive temporal shift that is consistent when assessed in the two halves of the data (Fig. 5, r=0.83, p=4×10^−4^). Applying an optimal temporal shift in these data appears strongly justified.

## 5 Discussion

The results presented in Fig. 3 suggest that pre-whitening primarily removes “false positive” associations between the model and data, i.e., when the nuisance regressors are hypothetically unrelated to the data. However, pre-whitening only reduced the voxelwise “false positives” from 51% to 13% (simulated model) and 34% to 9% (incorrect subject model), not reaching the expected 5% chosen as our *p*-value threshold. This is potentially due to two factors: firstly, the pre-whitening may not have been optimal. We tested the pre-whitened residuals produced in 3dREMLfit for remaining autocorrelation using the Durbin-Watson statistic, and observed that no brain voxels in the whitened residuals demonstrated evidence for positive autocorrelation. However, there was statistical evidence for *negative* autocorrelation in 4.5±0.05% of brain voxels (mean and standard deviation across subjects). This suggests that the ARMA(1,1) model used in this pre-whitening procedure did not optimally describe the resting state intrinsic brain fluctuations in all voxels, and future work to determine optimal pre-whitening for these data may be worth pursuing.

The apparent false positive rate may also exceed 5% because of non-zero correlation between the simulated nuisance regressors and the true regressors, and between the true regressors of different subjects. As presented in Supplementary Figure 2 in (Bright and Murphy, 2015), the noise model from another subject shares variance with the true noise model by chance; we therefore expect the model to explain significant variance in the data more frequently than chance. Thus, we contend that the observed rate across all brain voxels of significant R^2^ for the noise model is reduced to a reasonable level following pre-whitening.

In addition to testing the fit of the total noise model, we also probed potential problems that arise in evaluating and optimising an individual nuisance regressor, using the P_ET_CO_2_ regressor as our test case. The literature provides compelling evidence for the relationship between P_ET_CO_2_ and the BOLD signal (Blockley et al., 2011, Kastrup et al., 2001, Posse et al., 2001, Zande et al., 2004), and measuring “cerebrovascular reactivity” to CO_2_ is an emerging tool in clinical imaging (Pillai and Zacá, 2011, Spano et al., 2013). The majority of such studies examine the response to large changes in P_ET_CO_2_ levels induced by breath-hold, gas inhalation, hyperventilation, or other respiratory challenges. Still, resting fluctuations in P_ET_CO_2_ have been observed as significantly correlated with the BOLD time-series (Wise et al., 2004), supporting the removal of this variance from resting state data via nuisance regression to remove vascular confounds in brain connectivity measures.

Despite these well-established physiological links, our results suggest that the relationship between the BOLD signal and P_ET_CO_2_ is not always robust in the resting state data. After Šidák correction of the significance threshold, only subjects 3, 5, 6, 11, and 12 demonstrate a significant correlation that also exceeds the relationship with simulated regressors. The breath-hold data, however, presents a much more straightforward picture: all datasets demonstrate significant correlation that peaks at a physiologically plausible (and consistent) temporal shift.

From these observations we conclude the following:

1. Established nuisance regressors may not significantly contribute to the BOLD signal time-series in all datasets. In such cases, including these regressors in the noise model may remove variance from the fMRI data at random, acting similarly to unrelated regressors with similar frequency content.
2. Temporal shifting of P_ET_CO_2_ regressors is merited. The “optimal shift” in the breath-hold data is consistently non-zero, and thus the P_ET_CO_2_ regressor should be shifted to remove the correct noise variance from the fMRI data. This is likely also true for other physiological regressors.
3. The optimal temporal shift may not be reliably identified in resting state datasets where there is weak correlation between the BOLD and P_ET_CO_2_ data. In several subjects, the optimal shift in the resting state data does not match the optimal shift identified in the breath-hold data. Furthermore, the optimal shift may result in negative correlation, although negative reactivity is not expected except in a small subset of voxels (Bright et al., 2013), or else there may be no clear optimal shift within a physiologically plausible range.
4. Validation of the optimal temporal shift should be applied to test whether shifting of the nuisance regressor is justified. Validation can be achieved by comparing the optimal shift obtained in subsets of the data: a significant correlation between repeated estimations of the optimal shift should be observed prior to applying that shift to a given nuisance regressor.

To summarise, the relationship between nuisance regressors and fMRI data should be routinely examined, even when there is ample evidence for a certain relationship in the literature (as is the case with P_ET_CO_2_). In addition, there are varied motivations for shifting or otherwise optimising a given nuisance regressor at the group, individual, or voxel level, but unless these optimisations are demonstrated to be statistically significant (with appropriate corrections) and appropriately validated they may result in increased fMRI variance being removed from the dataset at random.

We have applied a simple validation technique at the individual subject level, comparing the results derived from the first and second halves of the average grey matter data from one fMRI dataset. Time-permitting, a second “training” dataset could be acquired to increase the degrees of freedom available in the analyses. A training dataset with amplified noise variance (e.g., breath-holds) would make the relationship between the nuisance regressor and fMRI signal more robust, and thus improve characterisation of any temporal lags. Here, we have used the correlation coefficient to validate the repeated measurements of the optimal temporal shift for the P_ET_CO_2_ regressor, however more rigorous cross-validation approaches may also be warranted. For example, metrics such as the Intraclass Correlation Coefficient (ICC) can test whether the optimisation of temporal shifts at the voxel level results in more or less reliable spatial maps of the correlation between the nuisance regressor and fMRI time-series across the study cohort (Bright and Murphy, 2013a, Shrout and Fleiss, 1979).

### 5.1 Functional connectivity

We have focused on how different statistical factors impact the process of nuisance regression, which aims to result in an accurately and sufficiently cleaned fMRI time-series that can be further analysed for functional connectivity. However, the pitfalls we have discussed are often problematic in connectivity analyses as well.

Similar to temporal shifting of nuisance regressors, sliding window analysis is often performed on resting state fMRI time-series to observe changes in connectivity over time (Hutchison et al., 2013). Although several groups apply rigorous statistical corrections to ascertain whether dynamic changes in connectivity are significant, these corrections are not universally adhered to, and this specific pitfall has been recently addressed in the literature (Shakil et al., 2016).

Because the cleaned fMRI time-series are highly autocorrelated (driving the aforementioned need for pre-whitening), the correlation between two of such time-series from unrelated brain regions will be inflated. Correcting for this autocorrelation prior to calculating correlation values has been tried, and although it did not significantly impact network results in healthy participants (Arbabshirani et al., 2014), it may impact quantitative comparison of connectivity metrics between cohorts with different inherent autocorrelation properties.

It was also proposed that functional connectivity measurements should be made on whitened residuals, rather than the “cleaned” time-series we have been discussing (Christova et al., 2011, Lewis et al., 2012). Whitened time-series are known as “innovations” to denote that they carry new information that is unrelated to previous time-points. These papers assert that correlations between innovations more accurately reflect the true underlying relationships between brain regions. After applying pre-whitening (modelling the BOLD signal as an ARIMA(15,1,1) process), the correlation between the innovations of 52 brain regions was calculated. This connectivity analysis revealed none of the “resting state networks” typically observed in the literature; hierarchical tree clustering revealed instead a functional organisation of brain regions that closely resembled cortical anatomy and showed strong links between homologous areas across hemispheres (Lewis et al., 2012). The authors present a strong and coherent argument for correcting non-stationarities and autocorrelations in BOLD time-series prior to calculating correlations between time-series, which we parallel here in the context of nuisance regression. We recommend that future connectivity studies consider the impact of autocorrelation on their connectivity metrics, whether by correcting correlation statistics or by analysing the whitened innovations present in the data.

### 5.2 Future work

Returning to the main motivation of this paper, it is generally beneficial to use the smallest sufficient noise model to avoid unnecessary reduction in the degrees of freedom in the fitting procedure. Although many of the contributing nuisance regressors in the resting state fMRI noise model are very well established, improved and potentially fewer regressors may be better for precise, accurate denoising.

Principal Component Analysis (PCA) of 4-D fMRI datasets generated using motion correction transformations has been used to create improved head motion regressors that may better reflect the nonlinear effects of movement during scanning (Patriat et al., 2016). PCA has also been applied to isolate the dominant signal fluctuations in regions of interest, such as white matter or ventricles, that are hypothesized to be dominated by noise. For example, in CompCor (Behzadi et al., 2007, Soltysik et al., 2015), multiple noise sources are described by a single nuisance regressor estimated from a subset of the fMRI data, which can substantially decrease the size of the noise model and potentially improve the accuracy of denoising.

A similar technique, ANATICOR (Jo et al., 2010) uses a local white matter region of interest to characterise multiple sources of signal noise in one nuisance regressor, which is tailored for each grey matter voxel across the brain. Adaptive noise models, where the specific nuisance regressors vary from voxel to voxel, are currently employed throughout the field. RETROICOR is typically applied using slice-specific temporal shifts to account for systematic delays in image acquisition in typical 2D EPI scans (Glover et al., 2000, Murphy et al., 2013). SLOMOCO provides slice-specific motion regressors that increase the accuracy of de-noising without relying on temporal shifts from known acquisition delays (Beall and Lowe, 2014). In dual echo acquisitions fMRI acquisitions, a short-echo time-series with minimal BOLD contrast can be applied as a voxel-specific nuisance regressor to remove head motion and cardiac pulsation noise (Bright and Murphy, 2013b, Buur et al., 2009). It may be desirable to further develop voxel-specific noise models, using both the data-driven and modelled nuisance regressors described in this paper. However. this could result in regional variations in the effective degrees of freedom in the fMRI time-series, for which we would need to account.

Similarly, it may be more appropriate to use expanded noise models in patient cohorts with large movement or physiological artefacts, while using reduced models in healthy controls; this would reduce the amount of interesting signal variance that was removed at random, but would also necessitate careful statistical compensation for the varying degrees of freedom in the resulting data. Such decisions to expand or reduce the noise model should be made in a systematic way, based on fitted parameter estimates. The field of model selection is extensively documented elsewhere, and is outside the scope of this paper. However it is important to consider the covariance across the many nuisance regressors included in the noise model, as some model reduction techniques assume independence of individual regressors.

We also observed that the ARMA(1,1) model did not fully pre-whiten the GLM residuals in this study, and more complex models may need to be used to identify the optimal pre-whitening method in denoising resting state data. Although the bias introduced by imperfect pre-whitening may not ultimately impact model fit estimates in task-activation fMRI (Marchini and Smith, 2003), it is yet unclear whether it is an important consideration in nuisance regression. Spatial smoothing of autocorrelation structure during pre-whitening is often applied in task-activation studies (Worsley, 2005); however this must be done carefully, as non-Gaussian spatial autocorrelations can drive false positives in further analyses of resting state data (Eklund et al., 2016). Finally, the application of bandpass filtering prior to GLM fitting effectively pre-colours the data, and as such, the pre-whitening techniques described here may no longer be appropriate.

## 6 Recommendations

Based on our observations and the literature referenced in this paper, we make the following recommendations:

• Pre-whitening should be applied during nuisance regression
  1. Existing pre-whitening tools in standard software packages are probably sufficient and can be easily applied, although further optimisation may be warranted
• If applied, temporal filtering should be incorporated into the GLM procedure
  1. Note, bandpass filtering prior to the GLM effectively ‘pre-colours’ the data, and pre-whitening techniques may no longer be appropriate.
• Nuisance regressors, particularly ones of physiologic origin, may need to be temporally shifted (or otherwise adapted) to best model the associated BOLD time-series
  1. The relationship between the regressor and data must be significant to obtain a robust estimate of the optimal temporal shift.
  2. The reproducibility of the optimal shift obtained from different subsets of the data should be used to validate the appropriateness of applying this shift.
• Nuisance regressors should be routinely assessed for significance and accuracy; in a given resting state dataset, well-established noise sources may not add significantly to the noise model and instead remove variance from the data at random.
• The specifics of pre-whitening, filtering, shifting, and associated statistical corrections during nuisance regression should be documented in manuscripts to foster consensus of methods across the field.
